# The crucial role of collagen-binding integrins in maintaining the mechanical properties of human scleral fibroblasts-seeded collagen matrix

**Published:** 2011-05-20

**Authors:** Shoulong Hu, Dongmei Cui, Xiao Yang, Jianmin Hu, Wenjuan Wan, Junwen Zeng

**Affiliations:** 1 Zhongshan Ophthalmic Center, State Key Laboratory of Ophthalmology, Sun Yat-sen University, Guangzhou, P.R. China; 2 National Key Discipline of Pediatrics (Capital Medical University), Ministry of Education, Department of Ophthalmology, Beijing Children’s Hospital, Capital Medical University, Beijing, P.R.China

## Abstract

**Purpose:**

The aim of this study was to identify the presence of collagen-binding integrin subunits in human scleral fibroblasts (HSFs) and investigate their actual functions in maintaining the mechanical creep properties of the HSFs-seeded collagen matrix.

**Methods:**

Primary HSFs were cultured in vitro. Reverse- transcription PCR was used to detect mRNA expression of integrin α1, α2, and β1 subunits in HSFs. In addition, western blot analysis and immunofluorescence were used to detect their protein in HSFs. Monoclonal antibodies were applied directly against the extracellular domains of integrin subunits in HSFs cultured in the three-dimensional collagen gels to block the interaction between HSFs and the extracellular collagen matrix. The effects of anti-integrin antibodies on HSFs morphology in collagen gel were observed. The effects of the added antibodies on fibroblast-mediated collagen gels’ contraction were evaluated. Furthermore, the changes in mechanical creep properties of collagen gel were measured by a biomechanics test instrument.

**Results:**

The mRNA and protein expressions of collagen-binding integrin α1, α2, and β1 subunits were present in HSFs. The elongated bipolar cells converted to spherical shapes after 6 h after the addition of integrin α1β1 and α2β1 antibody. The blocking of integrin α1β1 and α2β1 subunits noticeably decreased the contraction in the collagen gels. In addition, all samples were subjected to a constantly applied load of 0.03 N for 600 s. The blocking of integrin α1β1 and α2β1 subunits also induced increases in the values of final extension, creep extension, and creep rate, compared to those of the controls (p<0.01). Furthermore, the creep elements were significantly increased with the augmentation of the integrin antibody dose (p<0.01). The final extension of the integrin α2β1 antibody (1 μg/ml or 4 μg/ml) group was significantly higher compared to that of the integrin α1β1 antibody (1 μg/ml or 4 μg/ml) group (p<0.01). However, the creep extension and creep rate of the integrin α2β1 antibody (1 μg/ml or 4 μg/ml) group were not significantly different from those in the integrin α1β1 antibody (1 μg/ml or 4 μg/ml) group (p>0.05).

**Conclusions:**

Our findings suggested that HSF integrin α1β1 and α2β1 participated in maintaining the mechanical creep properties of the HSFs**-**seeded collagen matrix. Furthermore, integrin α2β1 might play a more crucial role in maintaining the mechanical creep properties of the collagen matrix than does integrin α1β1.

## Introduction

Myopia is a common ocular problem that affects perhaps one billion people worldwide [1].Most myopia is produced by lengthening of the ocular globe [2]. Much study has shown that the axial eye length can change with intraocular pressure (IOP). Congenital glaucomatous eyes show an increased axial eye length, whereas decreased axial eye length with lowered IOP is seen after trabeculectomy [3-5]. Given the evidence, progressive myopia is thought to result from an inherited biomechanical weakness of the sclera that allows it to stretch (creep) in response to stress [6]. Further evidence suggests that the biomechanical properties of the sclera may play a significant regulatory role in the axial elongation of myopic eyes. Creep describes the slow, time-dependent extension (or compression) of a sample of material when a constant load is applied (i.e., extension versus time). Studies of changes in the creep properties of sclera in myopic eyes imply that the posterior sclera from tree shrew eyes with induced myopia has a higher creep rate than does that from normal eyes. In contrast, samples from eyes recovering from induced myopia have decreased creep rates. Moreover, creep rate appears to be modulated in parallel with increased and decreased rates of axial elongation, which indicates that the regulation of the time-dependent mechanical properties of fibrous mammalian sclera plays a role in controlling the axial elongation rate [7,8]. Numbers of articles have demonstrated that the sclera is not a static container of the eye, but rather is a dynamic tissue, capable of altering the composition of the extracellular matrix (ECM) and its biomechanical properties to regulate ocular size and refraction [1,9,10].

Integrins are a large family of heterodimeric membrane glycoproteins that play important roles in numerous cellular processes involving cell-ECM and cell-cell interactions. Integrins consist of one α and one β subunit forming a noncovalently bound heterodimer. Integrins have an additional inserted domain (αI domain) in their α subunit. Four out of the nine αI containing integrins, namely α1β1, α2β1, α10β1, and α11β1, are receptors for collagens [11]. Collagen-binding integrins were reported to play a crucial role in maintaining the structural and mechanical properties of the collagen matrix in skin tissues [12]. Previous studies suggested that collagen-binding integrins might be involved in the development of myopia [13,14]. However, little information is available concerning integrin expression in the sclera, especially in human sclera. Furthermore, it is unclear how integrins participates in maintaining the ECM’s mechanical properties. This fact encouraged us to examine the presence of major collagen-binding integrin subunits in human scleral fibroblasts (HSFs) and to investigate the relationship of the interaction between HSFs and the extracellular collagen matrix with contraction and mechanical creep properties of HSF-seeded collagen gel.

In the present study, we evaluated the expressions of collagen-binding integrin subunits in HSFs and their role in maintaining the creep properties of the collagen matrix, in an attempt to highlight the actual functions of collagen-binding integrin subunits in HSF-ECM interactions.

## Methods

### Tissue source

This study was approved by the Ethics Committee of Sun Yat-sen University (Guangzhou, China) and it complied with the tenets of the Declaration of Helsinki for biomedical research involving human tissue. Five healthy human eyes from donors (age range: 18–23 years) were obtained from the Eye Bank of the Zhongshan Ophthalmic Center (Sun Yat-sen University). All procedures related to animal operations in this study followed the Guide for the Care and Use of Laboratory Animals of the USA National Institutes of Health.

### Human scleral fibroblast isolation, culture, and identification

As previously described, primary HSFs were isolated from human donor eyeballs [15]. The cells were cultured in growth medium consisting of Dulbecco’s modified Eagle's medium plus Ham’s nutrient mixture F-12(DMEM/F12; Gibco, Grand Island, NY), 10% (v/v) fetal bovine serum, 100 U/ml penicillin/streptomycin (Invitrogen Corp, Carlsbad, CA). The cells were incubated at 37 °C in a 5% CO_2_ humidified incubator and media was changed twice a week. After reaching near confluence, the cells were subcultured at a split ratio of 1:3. Fibroblasts from the third passage to the sixth passage were used in this study.

### Reverse transcription-polymerase chain reaction

Reverse-transcription PCR was used to confirm the presence of specific mRNAs in human scleral fibroblasts. Total RNA was extracted from the fibroblasts with Trizol reagent (Invitrogen Life Technologies, Grand Island, NY) and confirmed using spectrophotometry and agarose gel electrophoresis. The RT step was performed at 42 °C for 60 min in a 20 μl solution containing 2.5 μg RNA, M-MLV 5× reaction buffer, 20 U of RNase Inhibitor, 0.5 μg oligo (dT) 18 primer, 1 mM dNTP Mix, and 200 U RNase-free reverse transcriptase according to the manufacturer’s instructions (Fermentas, Burlington, Canada). The nucleotide sequences of the primers used in the experiments and the GenBank accession number of the underlying sequences were denoted in Table 1. Each PCR was performed in a 25 μl solution containing 1 μl reverse-transcription reaction products, 2.5 μl 10× EX Taq buffer, 0.2 mM dNTP Mix, 10 pmol of upstream primer, 10 pmol of downstream primer and 2.5 U Taq DNA polymerase according to the manufacturer’s instructions (TaKaRa, Kyoto, Japan). The PCR program was 3 min at 95 °C, followed by 25 cycles of 30 s at 94 °C, 45 s at 60 °C (α1 and β1) or 55 °C (α2), and 45 s at 72 °C, followed by a final extension of 10 min at 72 °C in a thermocycler (Whatman Biometra, Goettingen, Germany). The PCR product was electrophorezed on 2% agarose gels containing 1 μg/ml ethidium bromide and then photographed under ultraviolet illumination (Alpha Innotech Corp, Santa Clara, CA). A standard DNA ladder was used as a size marker. Furthermore, the PCR products were sequenced (ABI 3730XL; Applied Biosystems, Carlsbad, CA).

**Table 1:** Primers used for reverse transcription-PCR amplification of human integrin subtypes.

**Gene product**	**GenBank accession**	**Forward primer (5′-3′)**	**Reverse primer (5′-3′)**	**Product size (bp)**
integrin α1	NM_181501.1	TCAAACGAGGCACAATTCTG	AGCAGGATGACCCATAATGG	280
integrin α2	NM_002203.3	GCCTTGCCTTAGGTAATCAG	CCAGGAATGCTGCTAAACAT	226
integrin β1	NM_033668.2	GAAAGACACATGCACACAGGAA	ACATGAACCATGACCTCGTTGT	180

### Indirect immunofluorescence

The HSFs were grown on slides in six-well plates until 70%–80% confluence occurred. The slides were fixed in 4% formaldehyde for 30 min at 4 °C after washed in PBS for three times and air-dried. The slides were washed with PBS three times again, covered with 10% normal goat serum diluted in PBS, and incubated for 20 min at 37 °C. The slides were then incubated at 4 °C overnight with the primary antibodies (anti-integrin α1, anti-integrin α2, and anti-integrin β1; Millipore Biotechnology, Billerica, MA) diluted at 1:100 in PBS. Controls for immunospecificity were included in all experiments, except PBS replaced the primary antibody. After washed with PBS, the slides were exposed to fluorescein isothiocyanate-conjugated (FITC) goat anti-mouse IgG antibodies diluted at 1:50 in PBS at 37 °C for 30 min. Then hoechst 33358 was added into slides for 5 min to stain the cell nucleus. Immunofluorescent images were taken using a confocal microscope (LSM 510 META, Carl Zeiss, Jena, Germany).

### Western blotting analysis

The monoclonal antibodies against integrin α1, α2, and β1 were obtained from Millipore Biotechnology (Billerica, MA). The cells were lysed in ice-cold modified radioimmunoprecipitation (RIPA) buffer at 4 °C for 30 min. Cell lysates were centrifuged at 13,000× g for 10 min to remove insoluble material. Protein concentrations were determined using the BCA method. Of the supernatant, 10 μg and 20 μg of each protein sample were separated by 12% sodium dodecylsulfate-PAGE (SDS–PAGE) and subsequently blotted onto polyvinylidene difluoride (PVDF) membranes (100 V for 1 h; Millipore), and the membranes were blocked in 5% fat-free milk (Santa Cruz Biotechnology, Santa Cruz, CA) at room temperature for 2 h while rocking. Membranes were incubated with anti-integrin α1, anti-integrin α2, and anti-integrin β1 in an incubation buffer containing 5% BSA overnight at 4 °C while rocking, after washing three times with TBST (Tris-base, sodium chloride, and Tween-20). The binding of the primary antibodies was revealed by horseradish peroxidase-conjugated secondary antibodies (Santa Cruz Biotechnology, Santa Cruz, CA). The proteins of the membranes were detected using an enhanced chemiluminescence immunoblotting detection system (Thermo Fisher Scientific, Rockford, IL) and the film was scanned.

### Preparation of collagen gels and contraction assay

Type I collagen was extracted by stirring adult rat tail tendons (from Sprague Dawley rats) for 48 h at 4 °C in a sterile 0.1% (vol/vol) acetic solution (300 ml for 1 g of collagen), and the resulting solution was centrifuged at 16,000× g for 1 h at 4 °C and stored at 4 °C [16].

In summary, HSFs were resuspended at 1×10^5^ cells/ml in DMEM/F12 (5% fetal bovine serum) containing 2 mg/ml collagen type I neutralized with 1 M NaOH. Monoclonal antibodies, each of which binds specifically to the extracellular binding domain of α1, α2, or β1 subunits, were purchased from Millipore Biotechnology. For the inhibition studies, anti-integrin monoclonal antibodies (α1+β1, α2+β1) or mouse anti-human IgG as controls (Invitrogen Life Technologies, Grand Island, NY) were incubated with the cells for 15 min at 37 °C, before their addition to the collagen solution. The anti-integrin monoclonal antibodies were used in the experiments at final concentrations of 1 μg/ml or 4 μg/ml and mouse anti-human IgG were used at final concentrations of 0.1 mg/ml. Mixture samples of 2 ml including HSFs, antibodies, and collagen solution were cased in the 12-well plates (Corning, Lowell, MA). These plates were pre-coated with sterile BSA (2% in PBS) overnight at 4 °C and were washed in sterile PBS before the studies. Following collagen polymerization for 1 h at 37 °C, the edges of the HSFs-seeded collagen gels were detached from the sides of the wells to give a floating gel, and culture medium that contained the propotional anti-integrin monoclonal antibodies or mouse anti-human IgG was added. The culture medium was changed every other day and the gels were incubated in culture medium at 37 °C for 72 h. The wells were photographed at 2 h, 4 h, 6 h, 8 h, 12 h, 24 h, 48 h, and 72 h. The surface area of the gels was determined by quantitative morphometry with Image-pro plus 5.0 (Media Cybernetics, Bethesda, MD) from the prints. The contracted surface area was expressed as a percentage of the initial area.

### Mechanical properties measured

To evaluate the mechanical properties of collagen gels, creep tests were performed at room temperature in air using dynamic mechanical analysis of materials and devices (ElectroForce 3200 test instrument; Bose Corporation, Eden Prairie, MN) on day 3. A double-bladed knife was used to cut 3 mm-wide by 5 mm-long collagen gel samples. In creep mode, a constant stress was set at 0.03 N for 600 s. Dynamic mechanical analysis was used to apply small, steadily maintained loads to collagen samples and to monitor the resultant extension over time. The 0.03 N load was applied gradually (0–0.03 N in 20 s) and held constant for 600 s while sample length was monitored. After application of the 0.03 N load, the rate of change of length of the samples took some time to stabilize. The extension at 600 s was considered as the final extension. The slope of the curve between 100 s to 600 s was taken as the creep rate. Creep rate was expressed as the creep extension over 100–600 s (in percent extension per hour).

### Effects of anti-integrin antibodies on human scleral fibroblasts morphology in collagen gel

Collagen solution (1 ml) containing HSFs prepared as described above was added to 24-well plates. After 24 h, a sufficient amount of fetal bovine serum-containing medium was added, and the collagen gel was gently detached from the dish to give a floating gel. The medium was removed, and one or two drops of anti-human integrin antibody solution (a mixture of anti-integrin α1 and β1 antibodies or a mixture of anti-integrin α2 and β1 antibodies diluted to 4 µg/ml with serum-free Dulbecco’s modified Eagle's medium) were added directly onto the collagen gel and mouse anti-human IgG (0.1 mg/ml) was as controls. Morphological changes of fibroblasts were monitored with an inverted microscope (T-B2.5XA; Nikon, Tokyo, Japan).

### Statistical analysis

Every experiment was repeated at least five times. Data was expressed as mean±standard deviation (SD). Statistical analysis was performed with software (SPSS version 13.0; SPSS, Chicago, IL) between two groups using a two-tailed Students’ *t*-test for unpaired values, and p<0.05 was considered statistically significant.

## Results

### The expression of integrin α1, α2, and β1 subtypes in human scleral fibroblasts

**Figure 1:**
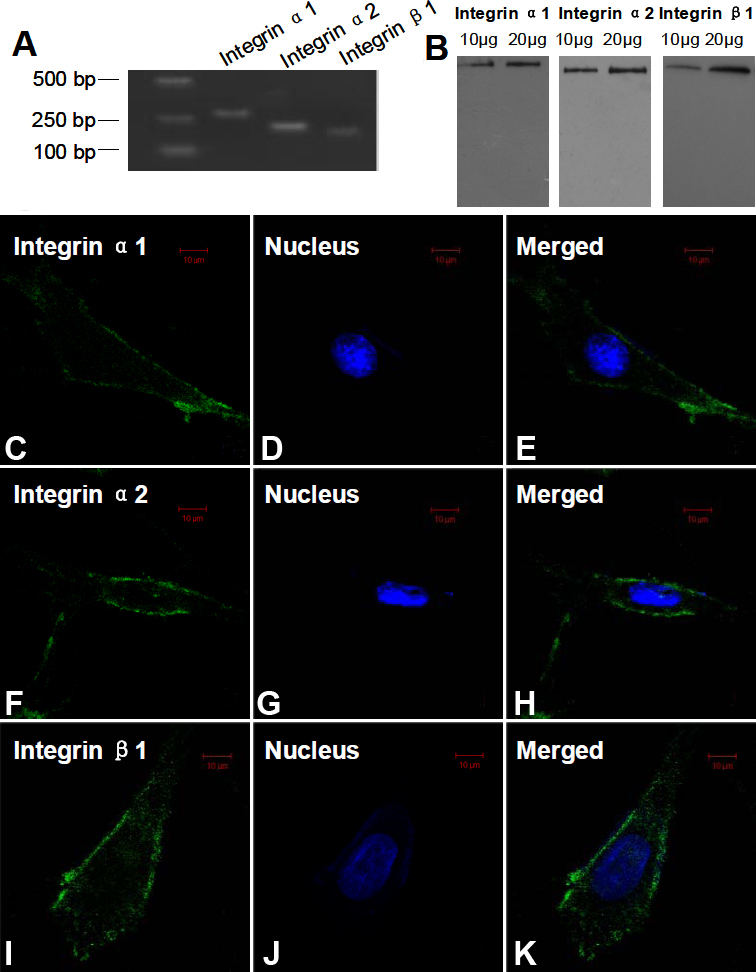
Identification of the collagen-binding integrins subtypes expressed in human scleral fibroblasts (HSFs). **A**: Amplification products representing the integrin α1 (280 bp), integrin α2 (226 bp) and integrin β1 (180 bp) subunits were detected in HSFs using reverse-transcription PCR. Molecular markers were included for product size comparison. **B**: The products representing the integrin α1 (130 kDa), integrin α2 (129 kDa), and integrin β1 (88 kDa) subunits were detected in HSFs using western blot analysis. **C**-**K**: Distribution of integrin α1, α2 and β1 in HSFs were observed by indirect immunofluorescence. FITC marked the secondary antibody (green; 1) and Hoechst33358 dyed the nucleus (blue; 2). The first (1) and second images (2) combined to form the third image (3). Integrin α1 (**C**-**E**), α2 (**F**-**H**), and β1 (**I**-**K**) were localized in the plasmalemma of HSFs.

Figure 1A showed that the mRNA expression of collagen-binding integrin subtypes (α1, α2, and β1) in HSFs was present. The predicted size of the products amplified by the PCR primers matched the size of the individual integrin subtype. Sequencing the amplicons confirmed that they truly were the intended product. Western blot analysis showed the presence of collagen-binding integrin proteins (α1, α2, and β1) in HSFs. Integrin α1 was detected as a 130 kDa band, integrin α2 as a 129 kDa band, and integrin β1 as a 88 kDa band (Figure 1B). In the indirect immunofluorescence, the fibroblasts treated with anti-primary antibodies showed green, and the nucleus showed blue (Figure 1C). Results from these tests indicated that the major collagen-binding integrins subtypes were present in the plasmalemma of HSFs.

### Effects of anti-integrin antibodies on human scleral fibroblasts morphology in collagen gel

**Figure 2:**
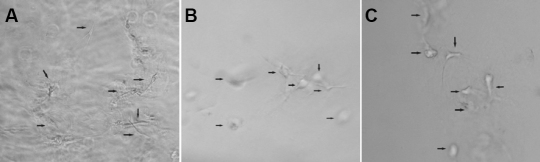
Morphological changes caused by the addition of anti-human integrin antibodies to human scleral fibroblasts (HSFs) in the collagen gel lattice. Anti-human integrin antibody (at the concentration 4µg/ml, which was diluted with serum-free DMEM) was added dropping it onto an HSF-populated collagen gel. Morphological changes of fibroblasts were monitored after 6 h addition using light microscopy (original magnification 200×). **A**: mouse anti-human IgG (0.1 mg/ml) was used as a control; **B**: A mixture of anti-human integrin a1 (4 µg/ml) and anti-human integrin β1 (4 µg/ml) antibodies were added. **C**: A mixture of anti-human integrin a2 (4 µg/ml), and anti-human integrin β1 (4 µg/ml) antibodies were added.

Initially, fibroblasts incorporated in collagen lattices were spherical in shape. By 6 h, cells in the collagen gel elongated and spread. After 24 h, all fibroblasts with elongated stellate/bipolar cell shapes were observed in the collagen gels. The shape changes were observed within several minutes after the addition of the antibody (α1β1, α2β1). The elongated bipolar cells converted to spherical shapes after 6 h (see Figure 2).

### Contraction assay

**Figure 3:**
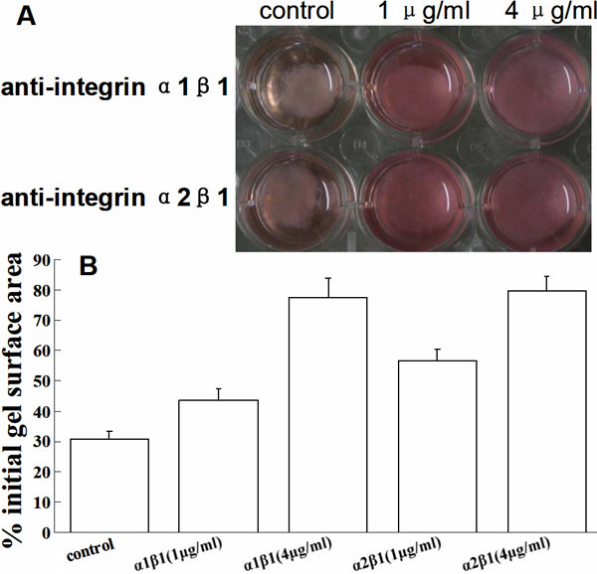
Gel contraction was dose-dependent inhibited by anti-integrin α1β1 and α2β1 antibody. Human fibroblasts were incubated in the absence (mouse anti-human IgG as control) or presence of a combination of anti-human integrin α and anti-human integrin β1 antibodies at final concentrations of 1 µg/ml and 4 µg/ml for 3 days. **A**: Representative photomicrographs show collagen gel changes. **B**: Contraction was indicated as percentage of the initial gel surface area.

In the control groups, contraction of the gels became visible 2–6 h after gel casting, being maximal between 12 h and 48 h, and reached a plateau at 72–80 h. Dose-dependent anti-integrin monoclonal antibodies inhibition of the contraction of collagen matrices were shown as a percentage of the initial gel surface area at day 3 (Figure 3).

### Mechanical creep properties of collagen matrix

**Figure 4:**
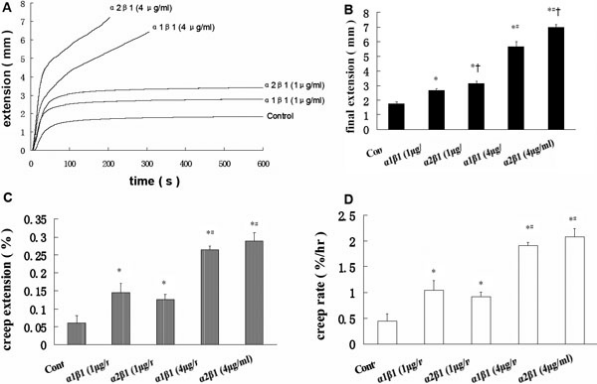
Anti-integrin a2β1 and anti-integrin a1β1 affect on mechanical creep properties of collagen matrix. **A**: Typical extension-versus-time behavior of the samples was shown, respectively. A constantly applied load of 0.03 N for 600 s was subjected to each sample. **B**: Anti-integrin a2β1 and anti-integrin a1β1 acted on the final extension of the HSF-populated collagen gel. **C**: Anti-integrin a2β1 and anti-integrin a1β1 acted on creep extension of the HSF-populated collagen gel. **D**: Anti-integrin a2β1 and anti-integrin a1β1 acted on the creep rate of the HSF-populated collagen gel. Results were expressed as mean±SEM *p<0.01 versus CON*;* ^#^p<0.01 versus α2β1 (1 μg/ml) or α1β1 (1 μg/ml); †p<0.01 versus α1β1 (1 μg/ml) or α1β1 (4 μg/ml).

Figure 4A showed typical extension-versus-time behavior of the samples. Samples were subjected to a constantly applied load of 0.03 N for 600 s. The extension at 600 s was considered as the final extension. Prior to that was the extension’s nonlinear phase (i.e., up to approximately 100 s), and the slope of the later, near-linear phase (approximately 100–600 s).

As shown in Figure 4B, the final extensions of controls, the integrin α2β1 antibody (1 μg/ml) group, and the integrin α1β1 antibody (1 μg/ml) group were 1.75±0.12 mm, 3.12±0.17 mm, and 2.66±0.13 mm, respectively. The final extensions of the integrin α1β1 antibody (1 μg/ml) group and integrin α2β1 antibody (1 μg/ml) group were significantly higher than that in controls (p<0.01). Furthermore, the final extension of the integrin α2β1 antibody (1 μg/ml) group was significantly greater than that in integrin α1β1 antibody (1 μg/ml) group (p<0.01). The final extensions of the integrin α2β1 antibody (4 μg/ml) group and integrin α1β1 antibody (4 μg/ml) group were 6.99±0.18 mm and 5.66±0.34 mm, respectively. The final extension was significantly increased with the augmentation of the integrin antibody dose (p<0.01). Furthermore, the final extension of the integrin α2β1 antibody (4 μg/ml) group was significantly higher, compared to that in the integrin α1β1 antibody (4 μg/ml) group (p<0.01).

In the following analyses, we considered extension during the near-linear phase (approximately 100–600 s) of the relationship to be a stable measure of creep extension. To isolate creep extension from the initial shorter-term changes in length, the absolute length of each sample was determined at 100 s after the load was applied. Creep extension (100–600 s) was computed as a percentage of this length. As shown in Figure 4C, the creep extensions of the controls, the integrin α2β1 antibody (1 μg/ml) group, and integrin α1β1 antibody (1 μg/ml) group were 0.06±0.02%, 0.13±0.01%, and 0.15±0.03%, respectively. The creep extensions of the integrin α2β1 antibody (1 μg/ml) group and the integrin α1β1 antibody (1 μg/ml) group were significantly higher than that of the controls (p<0.01). The creep extensions of the integrin α2β1 antibody (4 μg/ml) group and integrin α1β1 antibody (4 μg/ml) group were 0.29±0.02% and 0.26±0.01%, which was significantly increased with the augmentation of the integrin antibody dose (p<0.01). However, the creep extension of the integrin α2β1 antibody group was not significantly different from that of the integrin α1β1 antibody group, regardless of low or high doses (p>0.05).

Creep rate was computed as percent extension per hour. As shown in Figure 4D, the creep rate of controls, the integrin α2β1 antibody (1 μg/ml) group, and the integrin α1β1 antibody (1 μg/ml) group were 0.44±0.15%/h, 0.91±0.09%/h, and 1.05±0.18%/h, respectively. The creep rates of the integrin α2β1 antibody (1 μg/ml) group and integrin α1β1 antibody (1 μg/ml) group were significantly higher than that of the controls (p<0.01). The creep rates of the integrin α2β1 antibody (4 μg/ml) group and the integrin α1β1 antibody (4 μg/ml) group were 2.08±0.16%/h and 1.90±0.07%/h, which were significantly increased with the augmentation of the integrin antibody dose (p<0.01). There was no significant difference in the creep rate between the integrin α2β1 antibody and integrin α1β1 antibody groups, regardless of low or high doses (p>0.05).

## Discussion

Previous studies found that fibroblasts can condense a hydrated collagen lattice to a tissue-like structure, which can mimic the real environment in vivo, allowing study of the underlying mechanisms resulting from cell-ECM interaction [16]. This phenomenon is thought to be related to tissue remodeling [17]. The sclera tissue contains approximately 90% collagen by weight, consisting predominantly of type I collagen in mammals. Therefore, HSFs were provided with a three-dimensional matrix consisting of collagen type I, to elucidate the underlying mechanisms resulting from HSF and ECM interaction. Furthermore, by using the monoclonal antibodies of the major integrin to block the interaction between the ECM and HSFs, we evaluated, for the first time, the quantitative changes in the creep properties of collagen gels with HSFs seeded in.

Integrins could have a pivotal effect on the interaction of HSFs and ECM, which regulate the mechanical properties of HSFs-seeded collagen matrix. Integrins α10β1 and α11β1 have been discovered quite recently, and seem to be involved in bone and cartilage. Their functions are not quite clear [11]. The role of the α1β1 and α2β1 integrins as the major cellular collagen receptors in fibroblasts has been well documented [11,18,19]. Thus, the present study was undertaken to elucidate the expression of integrin α1, α2, and β1 in HSFs. In previous animal studies, the α1, α2, and β1 subunit expression decreased during the development of myopia, which showing that they may have positive regulator roles in the biomechanical remodeling that accompanies myopic eye growth [13]. By blocking interactions between collagen and HSFs with anti-integrin α1β1 and α2β1 antibodies, we focused on the changes in the contraction and the mechanical creep properties of the collagen, which are involved in the sclera remodeling related to myopia [7,8]. In all cases, the inhibition of gel contraction was correlated directly with integrin antibody concentration, suggesting that inhibition of collagen contraction is a specific effect caused by these integrin antibodies. The blocking of integrin α1β1 and α2β1 subunits induced increases in the values of final extension, creep extension, and creep rate, which reflected the creep elements of the collagen matrix. This was similar to an investigation that enhanced contraction result in the higher-strength collagen gels [17]. These changes in collagen contraction and mechanical properties suggested that the interactions between HSFs and collagen via integrins α1β1 and α2β1 regulated the mechanical properties of HSFs-seeded collagen matrix in vivo.

Similar differences between the functions of integrin α1β1 and α2β1 heterodimers in vitro have also been described. Integrin α1β1 was reported to regulate collagen synthesis and promote cell growth [20], whereas integrin α2β1 was a functional cellular receptor for type I collagen fibrils, mediating collagen gel contraction and regulating MMP-1 expression [21-23]. In our study, the inhibitions of the collagen gels’ contraction were more obvious from blocking interactions between collagen and HSFs with anti-integrin α2β1 antibodies than doing so with anti-integrin α1β1 antibodies. Furthermore, the blocking of integrin α2β1 subunits induced more significant increases in the values of final extensions, compared to the blocking of integrin α1β1 subunits. Our data suggested that α2β1 integrins in HSFs might be the major receptors responsible for regulating ECM remodeling.

Several studies have shown that antibodies against integrin inhibit cells from attaching to collagen type I [13,14,24-26]. In our study, the elongated bipolar cells converted to spherical shapes after the addition of the antibodies (α1β1 and α2β1). The morphological changes in HSFs indicated that the blocking of integrin subunits resulted in the detachment of fibroblasts from collagen, and that then the changes in mechanical properties might occur in vivo.

Based on the results of the present study, we consider human collagen matrix remodeling might be regulated through cell attachment to matrix molecules by integrin. The HSFs were inhibited from attaching to the collagen by blocking the collagen-binding integrins, so that the contraction of collagen matrices was inhibited, then collagen matrices lost their network structure and the mechanical proprieties changed. The present results might serve to explain the important observations that the integrin α1, α2, β1 subunits expression in sclera decreased during the development of myopia. That is, the sclera might become more extensible, resulting in its inability to resist the mechanical forces exerted by IOP. The eye thereby became longer, resulting in an axial myopia.

In summary, our present work provides evidence that the interactions between fibroblasts and collagen via integrins α1β1 and α2β1 regulate the mechanical creep properties of collagen matrix. Our data also underscore the possibility that α2β1 integrins in HSFs might be the major subunit responsible for regulating ECM remodeling. Further studies are needed to elucidate the exact role of collagen-binding integrins in myopia.
